# Tool-Wear-Estimation System in Milling Using Multi-View CNN Based on Reflected Infrared Images

**DOI:** 10.3390/s23031208

**Published:** 2023-01-20

**Authors:** Woong-Ki Jang, Dong-Wook Kim, Young-Ho Seo, Byeong-Hee Kim

**Affiliations:** 1 Department of Smart Health Science and Technology, Kangwon National University, 1 Kangwondaehak-gil, Chuncheon 24341, Republic of Korea; 2 Electric Power Train R&D Department, Korea Automotive Technology Institute, 303 Pungse-ro, Pungse-myeon, Dongnam-gu, Cheonan 31214, Republic of Korea; 3 Department of Mechatronics Engineering, Kangwon National University, 1 Kangwondaehak-gil, Chuncheon 24341, Republic of Korea

**Keywords:** tool wear estimation, deep learning, laser reflectance, monitoring system, IR vision system

## Abstract

A novel method for tool wear estimation in milling using infrared (IR) laser vision and a deep-learning algorithm is proposed and demonstrated. The measurement device employs an IR line laser to irradiate the tool focal point at angles of −7.5°, 0.0°, and +7.5° to the vertical plane, and three cameras are placed at 45° intervals around the tool to collect the reflected IR light at different locations. For the processing materials and methods, a dry processing method was applied to a 100 mm × 100 mm × 40 mm SDK-11 workpiece through end milling and downward cutting using a TH308 insert. This device uses the diffused light reflected off the surface of a rotating tool roughened by flank wear, and a polarization filter is considered. As the measured tool wear images exhibit a low dynamic range of exposure, high dynamic range (HDR) images are obtained using an exposure fusion method. Finally, tool wear is estimated from the images using a multi-view convolutional neural network. As shown in the results of the estimated tool wear, a mean absolute error (MAE) of prediction error calculated was to be 9.5~35.21 μm. The proposed method can improve machining efficiency by reducing the downtime for tool wear measurement and by increasing tool life utilization.

## 1. Introduction

Tool wear is one of the primary causes of poor machining quality. In severe cases, it can lead to tool and product damage, as well as unexpected machine downtime and failure [1]. As a result, a significant issue in the machining industry is the cost incurred by the frequent tool replacements required to ensure the reliability and stability of the manufacturing process and maintain product quality. Indeed, tool failures account for 7–20% of total operation downtime, and tool replacement costs account for 3–12% of total handling cost [2]. Therefore, tools are generally replaced before they are damaged to prevent failure; the replacement time is typically determined using a uniform-period replacement cycle based on the operator’s experience [3]. As the duration of this replacement cycle is inevitably conservative to prevent significant losses, only 50–80% of the life of a typical tool is used [4]. A tool-condition-monitoring system capable of directly and routinely managing tool wear is therefore required to reduce operational downtime while improving product quality [5,6]. An efficient tool-condition-monitoring system can also reduce machining costs by 10–40% [7,8]. To provide such monitoring, tool wear and remaining life must be accurately determined using appropriate measurement, signal processing, and data analysis techniques. Tool-wear-monitoring methods include direct measurement methods and indirect measurement methods. AI technology is actively utilized in various methods in the manufacturing industry [9,10,11], and various methods using AI technology in direct and indirect measurement methods are being studied for tool wear monitoring [12]. The direct measurement method is a method of directly measuring the tool wear area using an optical microscope or vision sensor. It shows high accuracy, but the cutting process must be stopped, and it can be measured while the tool is disconnected [13,14]. Indirect measurement methods are easy to implement by estimating tool wear by measuring signals such as cutting force, vibration, noise, and temperature changes with sensors during cutting. However, they must be measured during cutting and are highly affected by cutting conditions and the environment. Actual cutting conditions vary widely depending on the processing target, and it is a very difficult task to identify and analyze signals related to tool wear from the signals measured during the cutting process [15,16,17]. Therefore, in this paper, we tried to solve this problem by measuring the reflectance of the tool wear area to solve the problem of machining stoppage, which is a disadvantage of the direct measurement method.

**Figure 1:**
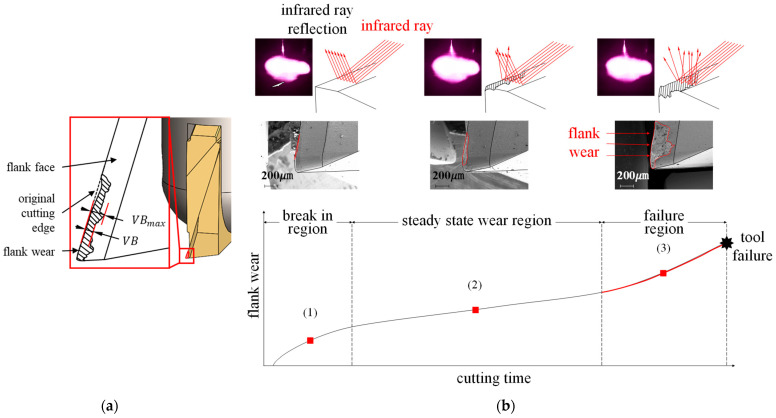
Tool wear: (**a**) Flank wear; (**b**) Tool wear curve.

Because the rotating tool is defined as one surface, all tools with a certain speed or higher can be applied, and the rotating state can be measured without separating the tool from the machine tool. Tool wear comprises a combination of wear types that occur when cutting, including fatigue wear, diffusive wear, plastic deformation, and adhesive and chemical wear. The combination of tool and workpiece materials employed exerts a critical influence on tool wear. Furthermore, even if the material combination is appropriate, wear characteristics may differ depending on the complex nonlinear relationships among the cutting parameters, workpiece, machine, and cutting tool characteristics, as well as the use/non-use of cutting oil and the cutting environment [2,8]. The two dominant types of typical tool wear are crater wear and flank wear. Crater wear refers to the wear of the rake face owing to its contact with the chip and is caused by the heat generated due to the friction between the two during cutting. This heat causes chemical reactions that spread between the tool and the material. Crater wear can occur if the spindle speed is too low or if the feed speed is too high [18]. Flank wear is caused by contact between the workpiece surface and the flank face, forming a wear land on the flank face of the tool through the abrasion of the machined surface and the tool blade during cutting [19]. Figure 1a depicts the flank wear of a tool. Flank wear has been widely used as a measure of general tool wear to establish the effective life of the tool. In the case of regular wear, a flank wear of *VB* = 300 μm is typically used as the standard for tool life. In the case of irregular wear, scratches, or severe grooves, *VB*_max_ = 500 μm is used.

Tool wear itself is defined according to ISO 8688-2 [20]. Figure 1b shows the increasing tool wear with ongoing usage time, the geometric change on the flank face of the tool, and the reflective characteristics of the tool surface according to this change. The tool wear process can be divided into the following three regions based on this trend: initial break-in; steady-state wear; and failure [21]. In the steady-state wear region, the coated tool surface geometry changes with ongoing wear according to usage time. Because the worn tool surface is rougher than the original tool surface, when a certain illumination is applied to the tool wear region, a degree of diffuse reflection occurs, corresponding to the degree of tool wear. In the failure region, the cutting temperature rises and the wear rate increases rapidly, eventually resulting in the end of tool life or tool failure [22]. Therefore, to prevent tool failure, an infrared (IR)-reflection-based tool wear imaging device was designed and fabricated in this study. The proposed device was then employed to collect images of steady-state flank wear from actual spinning tools. These images were processed using a variational autoencoder, and the results were applied to inform the estimation of tool wear using a multi-view convolutional neural network (CNN). In this study, tool infrared reflection images and multi-view CNN (convolutional neural network) to propose a method for estimating tool wear while mounted on a machine tool without stopping or removing the tool as in the conventional method. Therefore, it is expected that the maximum life span of the tool without damage to the workpiece can be used, and the machining time can be reduced.

## 2. IR-Reflection-Based Tool-Wear-Measurement Method

This section describes the experimental setup of IR-based image measurement equipment that can measure tool wear, how to measure images, and data preprocessing for CNN.

### 2.1. Fabrication of IR-Reflection-Based Tool-Wear-Measurement Device

**Figure 2:**
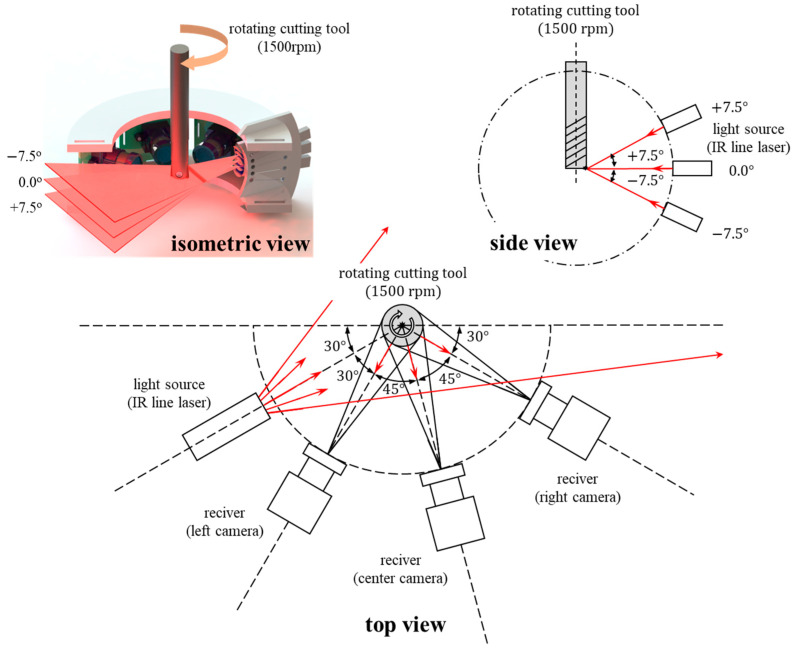
Proposed IR-reflection-based tool-wear-measurement device.

Figure 2 shows the configuration of the IR reflection measurement device proposed in this study. The center of the tool was set as the focal point for the IR light source, and an IADIY LM9IR940H50/L IR line laser with a laser light source was placed to irradiate the focal point at angles of −7.5°, 0.0°, and +7.5° relative to the vertical plane (parallel to the tool axis).

Tool face images were collected using ELP-USB13M02-V100 IR cameras with IR pass filters (Instrument Plastic OPTIR 1.0 NG) to minimize bleeding of the reflected IR light. Three such IR cameras were placed at equal intervals of 45° around the cutting tool with the laser light source placed at a 30° angle to the first IR camera, thereby measuring the reflected IR light from multiple angles. Thus, the fabricated measurement device could capture IR reflection images as the tool rotated.

### 2.2. Experimental Setup

**Figure 3:**
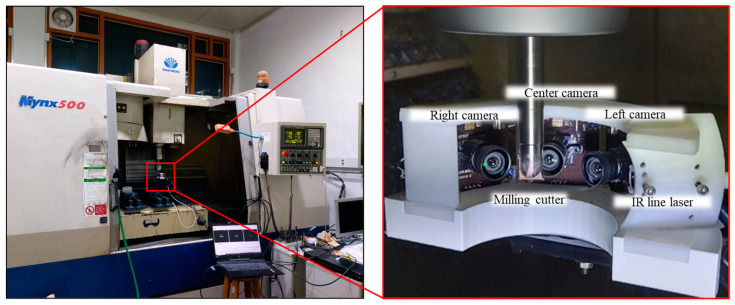
Experimental setup.

Figure 3 shows the fabricated measurement device and experimental setup. A DAEWOO Mynx 500 was employed as the machining center, inside which the proposed IR-reflection-based tool-wear-measurement device was installed.

**Figure 4:**
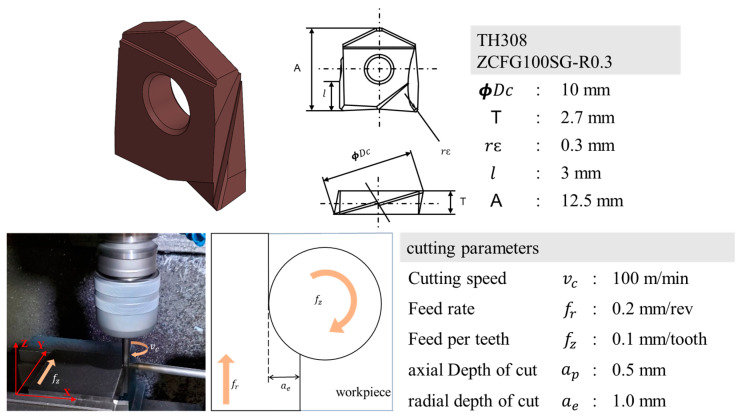
Information of milling insert and cutting processing parameters.

The tool used in the experiment was TH308 ZCFG100SG-R0.3 (Mitsubishi Hitachi). Figure 4 shows the information of the milling insert cutter and experimental cutting conditions, which were determined by referring to the manufacturer’s recommendations for gradual wear. The cutting speed ($v_{c}$), feed rate ($f_{r}$), feed per tooth ($f_{z}$), and radial depth of cut ($a_{e}$) when machining was set to $v_{c}=100\;m/\min$, $f_{r}=0.2\;\mathrm{mm}/\mathrm{rev}$, $f_{z}=0.1\;\mathrm{mm}/\mathrm{tooth}$, and $a_{e}=1.0\;\mathrm{mm}$, respectively. The axial depth of cut $a_{p}$ was changed arbitrarily, considering the cutting speed and feed rate. If the manufacturer-recommended value for $a_{p}$, 0.1 mm, was selected, the flank wear would be the same as the nozzle radius of the tool, making it difficult to form wear on the tool’s marginal surface. Therefore, an $a_{p}$ value of 0.5 mm, five times larger than the recommended cutting condition, was selected to produce sufficient wear. In the experiments, dry machining with the downward cutting of a 100 mm × 100 mm × 40 mm SDK-11 workpiece was performed while monitoring tool wear. Downward cutting was selected because the wear rate of the tool is faster in downward cutting than upward cutting. Table 1 shows the chemical composition of the workpiece.

### 2.3. IR Reflection Image Collection and Wear Measurement

**Figure 5:**
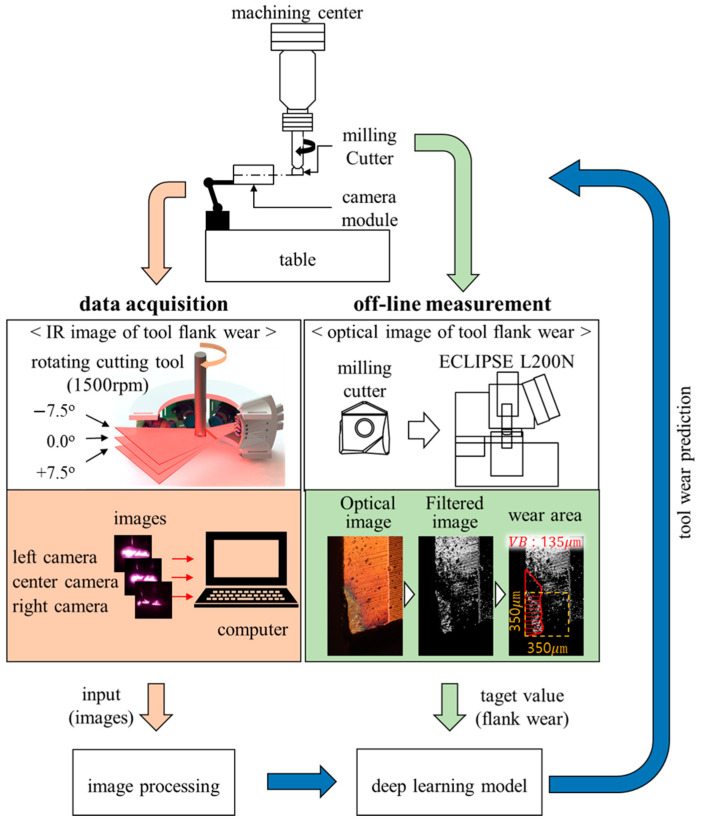
Schematic diagram of the proposed method.

Figure 5 presents a schematic diagram of the entire proposed tool-wear-measurement system. Workpieces with the tool were machined only up to 80 m because the tool’s corner would be damaged if machined to 85 m under the selected cutting conditions. An IR reflection image was measured every 5 m of cutting the length by idle-rotating the tool at 1500 rpm while adjusting it to the center of the proposed measurement device. Exposure times of 640 ms, 320 ms, 160 ms, 80 ms, or 40 ms were used to simultaneously collect 300 images each from the left, center, and right cameras, indicating a total of 900 images for each exposure time. After the IR reflection images of each worn tool were collected, the tools were disconnected from the machining center and photographed using a Nikon ECLIPSE L200N optical microscope to capture images of its flank wear. To ensure accurate measurement of tool flank wear, these images were converted into grayscale to remove color information, subjected to histogram equalization to improve contrast, and subjected to blur treatment to remove noise. The boundary of the wear region was then extracted from the processed images, and the flank wear width was measured 10 times at equal intervals in a 350 μm square box enclosing the tip of the tool. These measured flank wear values were averaged to obtain the actual tool flank wear.

### 2.4. Data Preprocessing

**Figure 6:**
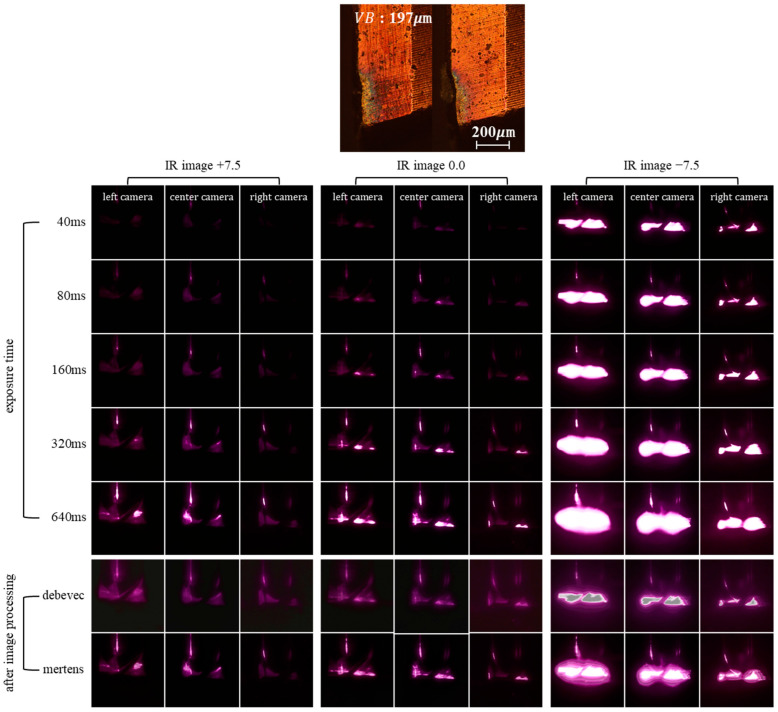
IR images of tool wear.

Figure 6 shows the reflected IR images for a measured tool flank wear of 197 μm according to exposure time and light source angles of −7.5°, 0.0°, and +7.5°. Note that these are low dynamic range (LDR) images, each of which clearly lacks a detailed representation of the reflection.

Indeed, unlike human eyes, cameras have a limited dynamic range of exposure. Because the actual scene captured in this study has a high dynamic range (HDR), but the camera captured only LDR images, they lack detailed representation in underexposed or overexposed areas. Thus, multiple LDR images were merged to create a single HDR image using a technique called HDR imaging. An effective technique for solving the problem of the limited dynamic exposure range of cameras, the HDR-imaging technique fuses multiple images of the same scene captured at different exposure levels [23,24]. To facilitate HDR-imaging applications, Debevec et al. [25] proposed creating HDR radiance maps by deriving the response function and relative radiance value from an image set captured at various exposures, whereas Mertens et al. [26] proposed a multi-exposure fusion method that generates a final fused image using images captured at different exposures according to their saturation and exposure quality. Thus, *N* weight maps W can be used to fuse images as follows [27,28]:

(1) $$W_{ij,k}={\left(C_{ij,k}\right)}^{\omega_{C}}\times {\left(S_{ij,k}\right)}^{\omega_{S}}\times {\left(E_{ij,k}\right)}^{\omega_{E}}$$

where $\left(i,\;j\right)$ refers to the pixel position in the image; k denotes the weight map number 1 to *N*; *C*, *S*, and *E* refer to contrast, saturation, and well-exposedness, respectively; and $\omega_{C}$, $\omega_{S}$, and $\omega_{E}$ denote the corresponding “weighting” exponents.

Using the weight maps calculated by Equation (1), the weighted average can be calculated for each pixel as follows [27,28]:

(2) $${\hat{W}}_{ij,k}={\left[\overset{N}{\underset{k^{\prime }=1}{\sum }}W_{ij,k^{\prime }}\right]}^{-1}W_{ij,k}$$

where the values of the *N* weight maps are normalized such that their sum will be 1 at each pixel.

The final fused image R can be obtained by [28]

(3) $$R_{ij}=\overset{N}{\underset{k=1}{\sum }}{\hat{W}}_{ij,k}I_{ij,k}$$

where $I_{ij,k}$ refers to the *k*th input image.

In this study, a single HDR image was created by merging the data from five IR reflection images collected at different exposures using either the Debevec or Mertens preprocessing methods to enhance the detailed representation of light.

## 3. Estimation of Tool Wear

This section describes processing a dataset of reflected IR images using a variational autoencoder (VAE) deep-learning model to achieve high classification performance for the proposed measurement method. Moreover, a method to estimate tool wear using multi-view CNN was explained.

### 3.1. Evaluation of Data Characteristics Using a Variational Autoencoder

**Figure 7:**
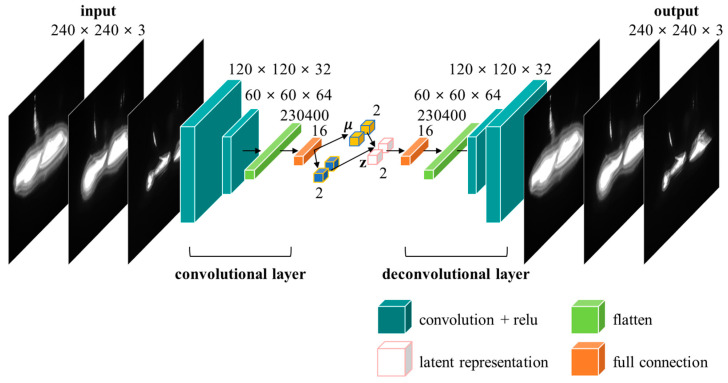
Variational autoencoder with convolutional layer architecture.

The latent vector of the VAE stresses the unique functionality of the given dataset, and the compressed latent representation can be used for visualization [29]. When the VAE model was trained using the IR image datasets, the latent spatial changes were used for data visualization and analysis. Figure 7 shows the detailed structure of the VAE used to evaluate the IR reflection images of tool wear. The Keras library, which uses Google’s TensorFlow library as the backend, was employed to construct the VAE model [30].

An autoencoder is a type of artificial neural network for unsupervised learning and is designed to compress input data into a lower-dimensional, latent representation space, then reproduce the compressed data as original data [30,31]. The VAE has a similar form to that of conventional autoencoders and comprises two neural networks: an encoder and a decoder. The encoder encodes the input data x into the latent representation *Z* [31,32] as follows:

(4) $$Z=g\left(x;\Phi \right)$$

where Φ and *x* are the parameters of the two neural networks.

The decoder reconstructs the latent representation *Z* as follows:

(5) $$\overset{ˇ}{X}=f\left(Z;\theta \right)$$

The VAE differs from other autoencoders in its use of Bayesian inference to create the latent representation of the data as implemented by

(6) $$Z_{i}=\mu_{i}+\sigma_{i}\cdot є$$

where $\mu_{i}$ and $\sigma_{i}$ are *i*th components of the mean and standard deviation vectors and є is a random variable following a standard normal distribution [33].

The cognitive network $g\left(\cdot ;\Phi \right)$ and the generative network $f\left(\cdot ;\theta \right)$ can be represented by $q_{\Phi }(Z|X)$ and $p_{\Phi }(X|Z)$, respectively, to obtain the following VAE loss function [26,31,32,33,34]:

(7) $$£\left(\theta ,\Phi ,X\right)=E_{Z\sim q_{\Phi }\left(Z|X\right)}\left[log_{\;}p_{\Phi }\left(X|Z\right)\right]-D_{KL}(q_{\Phi }\left(Z|X\right)||p\left(z\right))$$

**Figure 8:**
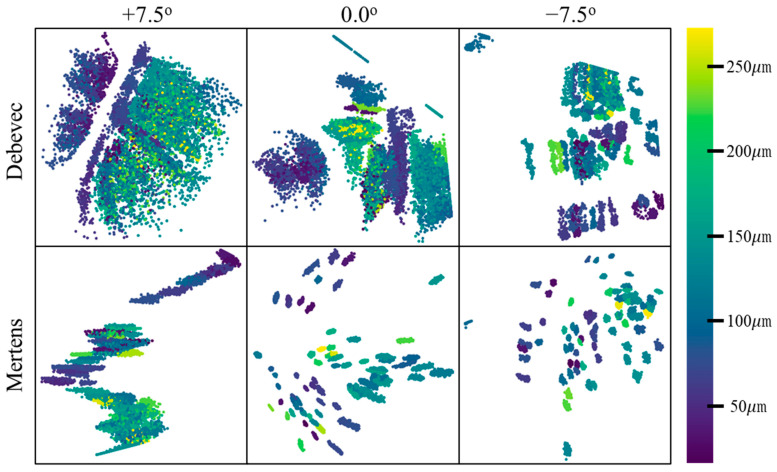
Dimensionality reduction results.

The reflected IR images measured from the left, center, and right cameras of the measurement device were converted into grayscale images after applying the Mertens or Debevec method. These converted images were stacked sequentially and used as a single input dataset comprising 240 × 240 × 3 images. The input data were compressed into two-dimensional vector form after passing through two convolutional layers comprising 32 and 64 filters. Next, the data were passed through the deconvolutional layers to generate a set of 240 × 240 × 3 images, which is the original form. Figure 8 shows the dimensional reduction results for the data compressed using the trained VAE model. When the dataset was clustered, a significant difference was observed between the results obtained using different data preprocessing methods, with the Mertens method clustering the dataset better than the Debevec method at all light source angles. Furthermore, the lower the position of the light source (i.e., as the light source angle decreased from +7.5° to −7.5°), the better the clustering. Indeed, the best clustering performance was obtained when the Mertens method was applied to the IR reflection image data measured using a light source angle of −7.5°.

### 3.2. Tool Wear Estimation Using Multi-View CNN

**Figure 9:**
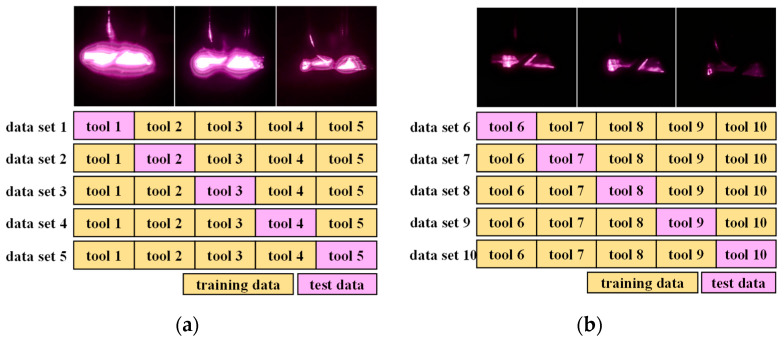
K-fold cross-validation method: (**a**) data without polarization filtering; (**b**) data with polarization filtering.

The data preprocessing and tool-wear-measurement method proposed using the VAE model were evaluated using the tool wear dataset compositions shown in Figure 9. In this evaluation, ten identical tools were used to conduct cutting exercises. The IR reflection images of tools 1–5 were collected from various angles using the device described in Section 2. Owing to the wide spread of the light source, the light was reflected from the worn regions of the tool, as well as other regions. Considering that the tool wear region was extremely smaller than the size of the entire tool, more light was captured from the non-worn region, causing bleeding when measuring the reflected IR light. To prevent this bleeding, near-infrared polarization filters were mounted on the light source and camera lens when collecting the reflected IR images of tools 6–10.

**Figure 10:**
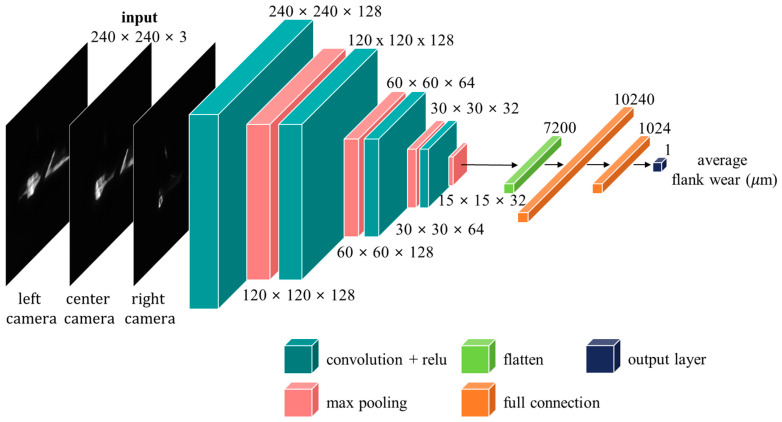
Deep-learning architecture for tool wear estimation.

The K-fold cross-validation method was employed to construct the training, evaluation, and test datasets. Thus, the data collected for tools 1–5 were used to derive five datasets for training and evaluation; in each dataset, one tool was used for the test data, while the remaining tools were used for the training data. The training and evaluation were performed for tools 6–10 in the same manner. Figure 10 shows the detailed architecture of the proposed multi-view CNN model for tool wear estimation. The input data first passed through the convolutional layers comprising 128, 128, 64, and 32 filters and pooling layers, resulting in a 15 × 15 × 32 dataset. The kernel sizes were 3 × 3 and 2 × 2. The result was transformed into a 1 × 7200 dataset to output the estimated tool wear. The fully connected layer was connected using 10,240 and 1024 nodes. As the proposed model was intended to output the tool wear in μm, a linear function was used as the activation function for the final output layer.

## 4. Results and Discussion

**Figure 11:**
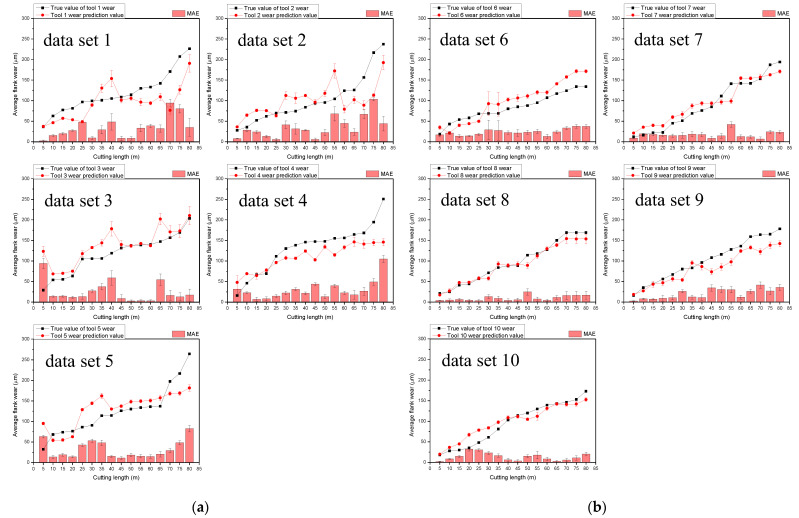
Tool-wear-estimation results: (**a**) data without polarization filtering; (**b**) data with polarization filtering.

As discussed in Section 3.2, four of the five tools in each dataset were used as training data, and the remaining tool was used as evaluation data; furthermore, 20% of the training data were used as validation data. As there were 4800 data samples measured for each tool, each dataset contained 15,360 training data points and 3840 validation data points. Figure 11a,b compares the measured tool wear with the estimated tool wear provided by the proposed deep-learning model without and with the polarization filters on the measurement device, respectively. The accuracy of the tool wear estimated by the CNN was evaluated in terms of mean absolute error (MAE) compared to the measured values, as shown in Table 2. The average MAE of the estimated tool wear for tools 1–5 (31.10) was larger than that of the estimated tool wear for tools 6–10 (16.84). Thus, the evaluation results indicated higher accuracy for tools 6–10, which had data collected using polarization filters mounted on the measurement device. The average MAE of the method to predict tool wear based on the cutting force signal using the autoencoder deep-learning model published in the existing literature was 34.93, and the maximum prediction error was less than 37 [17]. However, the method proposed in this paper had an average of 16.84 and a maximum prediction error of less than or equal to 23.67. In the case of average error, the performance improved by about 52%, and the maximum prediction error improved by 34%.

## 5. Conclusions

This study developed a novel tool-wear-measurement device using an inexpensive IR line laser and cameras to monitor tool wear faster and more accurately by employing deep learning. A predictive model was proposed to estimate tool wear based on reflected infrared images using the multi-view CNN algorithm, and the conclusions were as follows.

- Developed a reflection infrared image-measurement device and presented a method to estimate tool wear through measurement of the surface IR reflection light of the tool in a rotating state.
- Data were collected in various exposure conditions using the tool-wear-measurement device over a short period of machining.
- The IR reflection images of the surface of the worn tools were subsequently preprocessed and converted into HDR images using Mertens’ exposure fusion method to improve their quality.
- Tool wear was estimated from the collected IR images using a multi-view CNN deep-learning algorithm, and the results were compared to microscope observations to confirm accuracy.
- The tool wear prediction result of the method proposed in this paper was 16.84 on average, and the maximum was a prediction error of less than or equal to 23.67; therefore, compared to previous studies, the performance improved by about 52% on average, and 34% on maximum prediction error.

The proposed tool-wear-estimation method represents a vision-based direct measurement technique that exhibits high accuracy by routinely measuring during the operation period without requiring tool disassembly. This is a notable contrast to indirect measurement methods that limit or significantly affect tool usage time or operating conditions. Indeed, as the proposed method observes the rapidly rotating tool, it can perform measurements in real time without needing to cease operations and separate the tool. As a result, the proposed tool-wear-estimation method represents an optimal approach for direct tool condition monitoring.

Notably, the proposed tool-wear-measurement system was developed under dry cutting conditions using specific tools—circumstances in which it exhibited excellent performance. However, the proposed method was not evaluated under wet conditions, which are common in most cutting applications. Therefore, the proposed measurement system must be applied in wet environments in a future study to improve its performance. Still, the results of this study indicate that the accuracy of the proposed system can increase the time between tool replacement owing to wear, suggesting that it can facilitate the maximum utilization of tool life.

## Figures and Tables

**Table 1:** Chemical composition of the SDK-11 workpiece used in the experiments.

Chemical Composition (wt%)								
C	Si	Mn	P	S	Cr	Mo	V	Cu
1.536	0.348	0.536	0.020	0.001	11.00	0.857	0.475	0.069

**Table 2:** Evaluation of experimental results.

	MAE					
Data without polarization filtering	data set 1	data set 2	data set 3	data set 4	data set 5	average
	33.48	35.21	24.79	30.16	31.88	31.10
Data with polarization filtering	data set 6	data set 7	data set 8	data set 9	data set 10	average
	23.67	17.06	9.50	20.32	13.63	16.84

## Data Availability

Not applicable.
